# Bioavailability Study of an Innovative Orobuccal Formulation of Glutathione

**DOI:** 10.1155/2016/3286365

**Published:** 2015-11-16

**Authors:** Daniela Buonocore, Matteo Grosini, Silvana Giardina, Angela Michelotti, Mariaelena Carrabetta, Antonio Seneci, Manuela Verri, Maurizia Dossena, Fulvio Marzatico

**Affiliations:** ^1^Laboratory of Pharmacobiochemistry, Nutrition and Nutraceuticals, Department of Biology and Biotechnology “L. Spallanzani”, University of Pavia, 27100 Pavia, Italy; ^2^Farcoderm Srl European Expertise Network for Wellness and Dermatology, San Martino Siccomario, 27028 Pavia, Italy; ^3^Laboratory of Pharmacology and Experimental Toxicology, Department of Biology and Biotechnology “L. Spallanzani”, University of Pavia, 27100 Pavia, Italy

## Abstract

Alteration of the ubiquitous thiol tripeptide glutathione (GSH) is involved in oxidative stress, which plays a role in ageing; consequently, GSH is closely related to this process characterized by progressive decline in the efficiency of physiological function and increased susceptibility to disease. When circulating GSH decreases, oral administration might be considered a therapeutic benefit. Unfortunately, due to the hydrolysis of the tripeptide by intestinal *γ*-glutamyltransferase, dietary glutathione is not a major determinant for its increase. Aim of this work was to evaluate improvement of GSH systemic availability testing, *in vitro* and *in vivo*, an optimized orobuccal fast-slow release formulation tablet containing pure stabilized GSH. *In vitro* evaluation of the penetration capability of the innovative GSH-release formulation showed that GSH was well absorbed by the reconstructed oral epithelium and its absorption has features of time-dependence. In addition, *in vivo* results, obtained from 15 healthy volunteers, were in favor of GSH level improvement in blood showing fast (after 30 and 60 minutes) absorption through oral mucosa. In conclusion, the intake of GSH formulated through optimized orobuccal fast-slow release tablets gave positive results in raising GSH blood concentration.

## 1. Introduction

The intracellular production of oxidant species, for example, reactive oxygen species (ROS), is elevated in many neurodegenerative diseases related to inflammation and mitochondrial dysfunction [1–3]. The related process well known as oxidative stress (OS) is defined as “an imbalance between oxidants and antioxidants in favor of the oxidants, leading to a disruption of redox signaling and control and/or molecular damage” [4]. OS has been implicated also in normal ageing, particularly in the first phase of ageing [5], and many neurodegenerative diseases including Parkinson's (PD) and Alzheimer's (AD) diseases [6]. In both these diseases, multifactorial processes are involved, including OS, inhibition of mitochondrial complex I, ubiquitin-proteasome dysfunction, and inflammation. Furthermore, a number of events may occur simultaneously after OS including alteration of glutathione metabolism and GSH related enzymes, such as glutathione transferases (GSTs) [7].

*Glutathione and Its Implication in Ageing*. Glutathione (GSH) is a ubiquitous thiol tripeptide (gamma-glutamyl-cysteinyl-glycine), which plays an essential role in many cellular processes. It is believed to be the primary intracellular antioxidant for higher organisms. When oxidized, it forms a dimer (GSSG), which may be recycled in organs having glutathione reductase (GR). GSH is involved in many important biological phenomena, including the role of the brain's capacity to scavenge ROS, free radicals or not (e.g., superoxide radicals, hydroxyl radicals, and peroxynitrites). In particular, protection against oxidative stress is related to the oxidation of GSH in mitochondria [8, 9]. Liver produces daily and distributes through the blood stream to the other tissues about 80% of the 8–10 grams of glutathione. GSH depletion is considered as one of the most important and early biochemical changes in PD insurgence, suggesting that not only is it a consequence of OS but it could also play an active role in PD pathogenesis [10]. Glutathione depletion can inhibit complex I, E1 ubiquitin ligase (E1), and proteasome activity. It can also exacerbate OS and activate the Jun kinase (JNK) pathway, leading to an inflammatory response [10]. Decreased concentrations of glutathione were reported in the ageing rat brain [11–13]. Also in humans, low concentrations of glutathione have been observed in plasma, erythrocytes, lymphocytes, and gastric mucosa in older compared with younger adults [14, 15].

*Glutathione Availability*. The intracellular level of glutathione in nonnervous system mammalian cells is in the range of 0.5–10 mM, normally over 99% in the reduced form (GSH); particularly in humans, it is typically around 1-2 mM, several hundred times greater than the level seen in plasma blood, which is about 2 *μ*M in normal condition [16]. In the brain, however, GSH levels are often found at concentrations of 1–3 mM [7]. In particular, when the plasma circulating GSH decreases, due to different factors and provoking its lower availability into different districts and cells, oral administration might be considered a therapeutic benefit. Unfortunately, due to the hydrolysis of the tripeptide by intestinal *γ*-glutamyltransferase, dietary glutathione is not a major determinant for its increase; in fact results, obtained in a study of GSH systemic availability, showed that it is not possible to enhance GSH level to a clinically beneficial extent even by the oral administration of a high single dose of 3 g of GSH [17]. Therefore, there is the need to increase the systemic bioavailability of GSH when it is orally administered, to make it more suitable for therapeutic benefit, for example, those regarding the central nervous system.

Based on the above-mentioned considerations, the aim of this study was to evaluate the improvement of GSH absorption and systemic availability by testing an optimized orobuccal fast-slow release formulation tablet containing pure stabilized GSH. For this purpose,* in vitro* and* in vivo* evaluations were carried out. The term orobuccal refers to a composition capable of immediately dissolving and releasing the active ingredient contained therein upon contact with the oral mucosa. In this manner, the active compounds may be directly absorbed in the oral mucosa, which is well supplied with both vascular and lymphatic drainage, thus bypassing the intestinal degradation [18]. The above is only applicable to the molecules that can pass through the oral mucosa; all the other components are swallowed and go into the stomach, where they can be degraded by the gastric acid. Fast-slow release formulation reduces the problems of absorption and bioavailability of these components without losing the advantages of orobuccal absorption. The term “fast-slow release” means the differentiated release of selected ingredients: some components are delivered in orobuccal fast release and others in enteric slow release, by the use of new technology. This technology is applied to the selected molecules that are coated with a food grade fat matrix: when swallowed, the gastroprotected micro granules of the coated ingredient are not destroyed by the gastric acid and reach the intestine undamaged, where they are assimilated following the physiological lipid absorption pathway. The term “stabilized” refers to glutathione, which is maintained in a reduced form without substantial cyclization. This stabilization may be affected by the addition of one or more agents providing a formulation together with GSH, which is capable of delivering native reduced glutathione.

## 2. Materials and Methods

### 2.1. Sigma-Aldrich Chemicals Supplied All Chemicals and Solvents Used in This Study

SkinEthic supplied Reconstituted Epithelium HOE/S/5 (batch number 122 011J-M 037) and maintenance medium (batch number 12 022B 1103). Arbor Assays supplied hemoglobin (Hb) assay kit.

### 2.2. *In Vitro* Evaluation of the Penetration Capability of Optimized Orobuccal Fast-Slow GSH Release Formulation through Reconstructed Oral Tissue

The aim of the* in vitro* study was to investigate the penetration capability of GSH integrated in an orobuccal fast-slow release formulation through reconstructed human oral epithelium (SkinEthic HOE/S/5, batch number 12 022B 1103). This specific tissue was made up of 0.5 cm^2^ epithelium reconstituted by air lift culture of transformed human keratinocytes TR146 (from a squamous cell carcinoma of the buccal mucosa) for 5 days in chemically defined medium (Maintenance Medium, batch number 122 011J-M 037) on inert polycarbonate filters. These transformed keratinocyte cells form an epithelial tissue devoid of* stratum corneum*, resembling histologically the mucosa of the oral cavity [18].

The tested sample was powder for tablets, each 220 mg of powder containing 50 mg of pure GSH. A quantity of powder just equal to the amount in a tablet (220 mg containing 50 mg of GSH, 5 mg/mL) was preliminarily dissolved in 10 mL artificial saliva (KCl 20 mM, KSCN 5.3 mM, NaH_2_PO_4_ 1.4 mM, NaHCO_3_ 15 mM, and C_3_H_6_O_3_ 10 mM) at 37°C for 1 hour. According to Chotaliya (2013) [19] absorbing buccal surface is about 200 cm^2^, with a great vascularization network. About the saliva volume, there is a nonhomogeneous saliva production of about 1500 mL during the day, with a mean saliva production in normal condition of about 10 mL/h until 250 mL/h under stimulation or 0 mL/h during sleeping [19]. Specific volumes of the above-described solution, corresponding to 10 mL of saliva per 200 cm^2^ of surface, were applied onto 2 tissue units (0.5 cm^2^) in replicate following a chronological order of experimental times T10 (10 minutes after product application); T20 (20 minutes after product application); T30 (30 minutes after product application). We evaluated the dose of the reduced glutathione absorbed and released by the tissue into the underlying medium during the time. Furthermore, at the end of the experimental period, treated tissue was used to determine its viability in order to evaluate any possibility of irritation of oral mucosa by MTT (methylthiazolyldiphenyl-tetrazolium bromide) colorimetric assay [20]. At the end of the experimental period, we also evaluated the amount of glutathione absorbed in the tissue structure that was not released. In order to evaluate the endogenous production of glutathione during the treatment and correctly determine the absorption grade, a tissue series was treated with N-acetyl cysteine, precursor of glutathione. Glutathione dosage, in all treated models, was carried out by high-performance liquid chromatography (HPLC Jasco LC900) using C18 column Zorbax ODS column 250 mm 4.6 mm, 5 mm (Agilent Technologies).

### 2.3. *In Vivo* Evaluation of GSH Systemic Bioavailability Using an Optimized Orobuccal Fast-Slow Release Formulation Tablet

The aim of the* in vivo* study was to evaluate GSH bioavailability using an optimized orobuccal fast-slow release tablet on a number of 15 healthy volunteers (females and males), weight 60 ± 5 Kg, aged between 20 and 40 years, belonging to the European population (white); they did not practice exhaustive exercise and they are not smokers; they declared that they did not get any other food supplements or drugs. Criteria for the definition of the number and the expected number are 95% confidence level; estimates of the prevalence level of GSH/GSSG 0.2; estimate of the desired precision 0.2 (20%); CI 1.96; expected number 15. Volunteers were earlier informed on the procedures to be followed in the study, which was conducted with their understanding and consent. Each procedure was drawn up in agreement with the Helsinki declaration adopted at the Eighteenth General Assembly of the World Medical Association (WMA), held in 1964 on ethical principles for medical research involving human subjects. An Independent Ethical Committee of the University of Pavia approved the work procedures.

*Supplementation Protocol.* Subjects were provided with an orobuccal fast-slow release tablet of pure GSH taken through an oral absorption way. The principal ingredients of the tablet, with nutritional or physiological effect, are reported in Table 1. Pure GSH forms a flaky powder, which retains a static electrical charge, due to triboelectric effects, which makes processing difficult. The powder may also have an electrostatic polarization, which is akin to an electret (elektr- from “electricity” and -et from “magnet”). Glutathione is a strong reducing agent, so that autooxidation occurs in the presence of oxygen or other oxidizing agents. Demopouolus and Ross (1993) [21] provided a method of manufacturing glutathione tablets and capsules by the use of crystalline ascorbic acid as an additive to reduce triboelectric effects, which interfere with high-speed equipment and maintaining glutathione in a reduced state. Ascorbic acid has the advantage that it is well tolerated and antioxidant and reduces the net static charge on the glutathione [21].

A preferred formulation includes 250 mg or more of reduced glutathione with at least equimolar ascorbic acid, to fulfill three functions: acting as a sacrificial nonspecific antioxidant during preparation and storage and after ingestion; reducing or neutralizing static electrical charge of glutathione powder, allowing dense packing of capsules; and acting as a lubricant for the encapsulation device. The ascorbic acid also maintains an acidic and reducing environment, which pharmaceutically stabilizes the glutathione molecule. Ascorbic acid is believed to form a charge couple with glutathione, which enhances penetration through cell membranes and reduces the tendency for the gamma-glutamyl and glycinyl residues to assume a cyclic conformation or to form an internal cyclic amide. The ascorbate thus complexes with the glutathione in solution to maintain a linear conformation. This linear conformation, in turn, sterically hinders the free cysteinyl thiol group. This steric hindrance stabilizes a free radical, which may be formed, and thus maintains the biological activity of glutathione.

The oral mucosa has been found to allow rapid and efficient uptake of glutathione into the blood. In contrast to the digestive tract, the significance of facilitated or active transport mechanisms in the oral mucosa is believed to be low; rather, a high concentration of glutathione in the oral mucosa is believed to permit passive transport of the glutathione through the cells or around the cells into the capillary circulation. Therefore, compositions intended for absorption through the oral mucosa are preferably of high purity, as contaminants may be absorbed similarly to glutathione, and as relatively small, uncharged molecules [21].

To assess the GSH systemic bioavailability each volunteer had one tablet of orobuccal fast-slow GSH release melting it in the oral cavity in about 1 minute. The* in vivo* study was developed in acute, otherwise evaluations carried out in short experimental times. A draw blood was carried out before absorption (basal time) and after 30 minutes (T30) and 60 minutes (T60) starting from complete tablet absorption. Analysis of GSH levels was executed on whole blood employing enzymology GSH recycling method [22], on the basis of conversion of GSSG to GSH by GR and NADPH and reaction with 5,5′-dithiobis-(2-nitrobenzoic acid). We analyzed whole blood because we considered that the endogenous disappearance rate (utilization rate) of GSH was about 25 *μ*mol/(kg*∗*h) in healthy adult humans and that this rate accounted for 65% of whole body cysteine flux [38.3 *μ*mol/(kg*∗*h)]. Among extrahepatic cells, the erythrocyte had a relatively high turnover rate for GSH. For example, the whole-blood fractional synthesis rate of GSH in healthy adult subjects was 65% per day, which meant that all the GSH was completely replaced in 1.5 days; this value was equivalent to 3 *μ*mol/(kg*∗*h) [23]. We also took into consideration that whole blood (mainly erythrocytes) contributed up to 10% of whole body GSH synthesis in humans and that extracellular concentrations of GSH were relatively low (e.g., 2 *μ*mol/L in plasma), due to the cysteine residue of GSH, which was readily oxidized in a nonenzymatic way to GSSG by electrophilic substances (e.g., free radicals and reactive oxygen/nitrogen species). Another important reason for using whole blood was that the acids that are commonly used in protein unfolding and precipitation induce an overestimation of the amount of GSSG in blood samples because GSH is oxidized to GSSG (5–15%). We prevented this error by applying an innovative derivatization process that added the thiol-alkylating agent N-ethylmaleimide (NEM) to whole blood [24]. Therefore, we considered it to be accurate to measure glutathione in whole blood and to normalize and express it per g of Hb.

Results are expressed as nmol of GSH per gram of Hb. Evaluation of Hb concentration was implemented with a colorimetric kit assay (*Arbor Assays*, Michigan, US).

### 2.4. Data Analysis

Descriptive statistics were expressed as means and standard deviation of the mean (SD). In the* in vivo* study, a repeated measure analysis of variance was used to test the within-subjects variation in GSH absorption over time. We tested the sphericity assumption (through Mauchly's test) and since it was violated, we used the multivariate approach (MANOVA, Wilk's test) to test the within-subjects effects. The level of statistical significance was set at a *p* value of <0.05 (^*∗*^ *p* value < 0.05; ^*∗∗*^ *p* value < 0.01). NS means not statistically significant.

## 3. Results and Discussion

### 3.1. *In Vitro* Study

**Figure 1:**
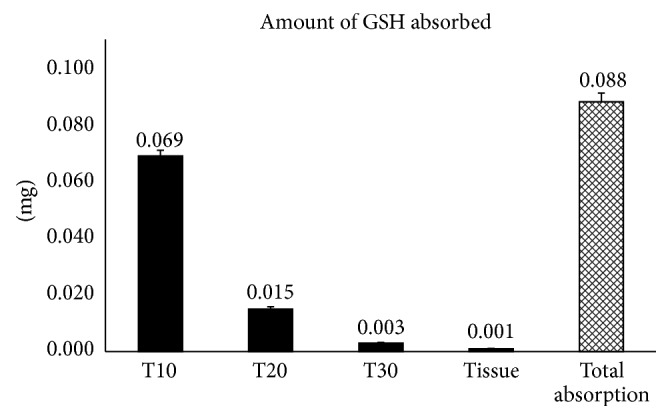
*In vitro* data obtained from GSH dosage as mg in the experimental model are reported. The measured glutathione amounts are expressed as mg (mean ± standard deviation SD) in the collected medium at different experimental times and in homogenized tissue at the end of experimental period. Total absorption at the end of the experimental period is also reported considering GSH amounts in the medium at different times and in the tissue.

We evaluated the dose of the reduced glutathione absorbed and released by the tissue into the underlying medium following a chronological order of experimental times (10, 20, and 30 minutes) and starting with applying 0.125 mg of GSH onto units of tissue. At the end of the experimental period, we also evaluated the amount of glutathione absorbed in the tissue structure that was not released (Table 2, Figure 1).

**Figure 2:**
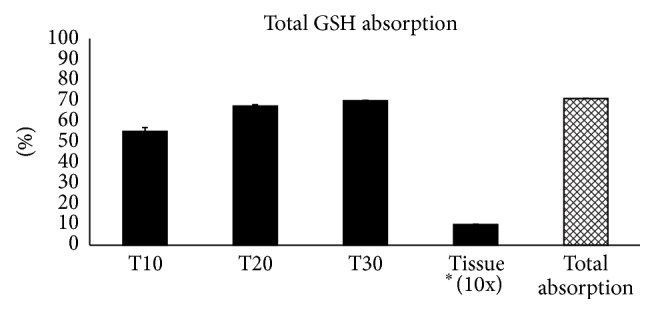
*In vitro* data obtained from the GSH dosage as % in the experimental model are reported. The percentage (%) of GSH absorption values are calculated on the applied amount of GSH (0.125 mg) and reported on the bases of single monitored experimental time as total % of absorption (mean ± standard deviation SD). Tissue ^*∗*^(%) value of GSH evaluated in the homogenized tissue is resized (10x).

The amount of GSH released into the medium decreased over time: 0.069 mg after 10 minutes, 0.015 mg after 20 minutes, and almost null (0.003 mg) after 60 minutes. These results showed that GSH within an orobuccal fast-slow release formulation was able to penetrate reconstructed human oral epithelium. Moreover, obtained data showed a very small capacity of the tissue to hold the absorbed glutathione, contributing to its bioavailability (Table 2, Figures 1 and 2). We found only 0.001 mg of GSH (100 parts less than the applied amount of 0.125 mg) in the homogenized tissue at the end of the experimental time.

Furthermore, the analysis of the obtained results showed not only a penetration capability but also a fast absorption of the glutathione through the* in vitro* reconstructed oral epithelium, from 55% after 10 minutes to about 70% after 30 minutes (Table 2, Figure 2). The kinetics of the passage through the epithelium mimic a progressive absorption gradient for glutathione, saturated over time.

According to the product classification reported on validated methods with EpiSkin or Reconstructed Human Epithelium (RHE), performed MTT test showed no sign of irritation for the oral epithelium after the application of the sample solved in artificial saliva (Table 3). The relative viability percentage (%) was calculated for test substance (treated tissue) compared to negative control (nontreated tissue) and compared to the following criteria: mean tissue viability is ≤50%: irritant (I); mean tissue viability is >50%: nonirritant (NI).

Collected experimental data showed that the GSH insight orobuccal fast-slow release formulation was absorbed by the* in vitro* reconstructed oral mucosa and its absorption had features of time-dependence. According to the* in vitro* data and to the considered experimental protocol, optimized orobuccal fast-slow GSH release formulation was capable of carrying the GSH for the absorption through the oral mucosa.

### 3.2. *In Vivo* Study

**Figure 3:**
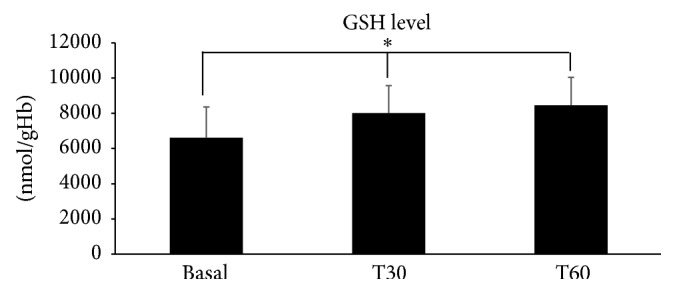
*In vivo *data obtained from the glutathione (GSH) dosage in whole blood are reported. Results are expressed as mean ± SD in nmol GSH per gram of Hb at different experimental times T30 and T60 versus basal (before starting orobuccal fast-slow release tablet absorption). Statistical analysis was carried out using repeated measures analysis of variance in order to compare the mean values at subsequent times. The GSH level increased significantly (^*∗*^ *p* = 0.014) with absorption time.

The analysis of the obtained results in acute study showed a fast absorption of the glutathione through the* in vivo* oral mucosa. GSH blood concentration was increased both 30 minutes and 60 minutes after taking the orobuccal fast-slow GSH release formulation compared to the basal time before starting absorption. This increase was statistically significant when comparing the baseline concentration levels and those of concentration after 30 and 60 minutes. It could therefore be inferred that GSH was actually absorbed through mucous membrane, in a rapid manner, going to increase the quantity of reduced glutathione present in the blood (as it was expressed in Table 4 and represented in Figure 3). Finally, obtained* in vivo* results suggested that glutathione, taken by tablets with orobuccal fast-slow release, showed good bioavailability. Optimized orobuccal fast-slow release tablet was able to increase GSH blood level.

*In vivo* study confirmed results obtained* in vitro*; in other words orobuccal fast-slow GSH release tablet showed to have a good and rapid absorption through oral mucous membrane. Furthermore, derivatization process with alkylating agent NEM [24] demonstrated to be efficient; in fact obtained GSH levels found correspondence with data presented in literature (e.g., considering Hb mean value of 0.5 g/mL, GSH concentration in cells has a range: 1000–20000 nmol/gHb).

Through orobuccal formulations, GSH is administered and absorbed directly in the oral cavity generating a much quicker absorption and a considerably higher bioavailability with respect to the current method of administration by means of gastroresistant tablets and considering the same dose of active ingredient. The orobuccal compositions may be formulated in form of tablet, capsule, and/or granules, preferably in form of orobuccal tablet. Due to high permeability, the oral absorption route can produce rapid onset of action so the “drug” with short delivery period can be delivered and dose regimen is frequent [18].

The interest in the studies of the mechanisms, which correlate and interfere with ageing, is ever growing. On one hand, oxygen is essential for aerobic organisms, because it is the final electron acceptor in the mitochondria; on the other hand, oxygen is harmful because it can generate ROS continuously, which is believed to be factors closely related to ageing. Aerobic organisms possess antioxidant defense systems, able to remove ROS in the cells. These systems consist of a series of enzymes and molecules of which the most effective is glutathione. Therefore, GSH plays a central role in the maintenance of cellular redox homeostasis and protection against ROS. Given the involvement of decreased GSH levels and alterations in glutathione-related enzyme activities in OS and related disease and regression states, maintaining or restoring GSH levels represents a promising “therapeutic” strategy. The apparently most straightforward approach for increasing GSH levels, through its direct administration, failed due to the solubility, absorption, and stability constraints that limit its bioavailability [25]. The direct delivery of the precursor amino acid cysteine was also a failure, due to its toxic effects [26]. Hence, research efforts turned to develop innovative GSH formulation to strengthen its cellular redox protective effects.

## 4. Conclusions

In conclusion, the intake of GSH, formulated through optimized orobuccal fast-slow release tablets, gave positive results in raising GSH blood concentration, which is probably going to strengthen all* in vivo* by-products and processes that involve this important tripeptide.

We can conclude that orobuccal fast-slow GSH release tablet is a new, innovative, efficient, and functional dosage form.

## Figures and Tables

**Table 1:** Orobuccal fast-slow release tablet.

Nutritional information		
Substance with nutritional or physiological effect	Daily dose (1 tablet 1.1 g)	%RDI (*∗*)
GSH	250 mg	
L-Cystine	50 mg	
Vitamin C	40 mg	50%
Selenium	55 mcg	100%

(*∗*) Recommended daily intake according to Dir.100/2008/EC.

**Table 2:** GSH absorption.

	GSH (mg)		GSH (%)		
Applied amount	0.125				

	Mean value	SD	Partial absorption	Total absorption	SD

T10 (medium)	0.069	0.002	55.04%	55.04%	1.9%
T20 (medium)	0.015	0.0008	12.32%	67.36%	0.63%
T30 (medium)	0.003	0.0002	2.56%	69.92%	0.14%
Tissue	0.001	0.0001	1%	1%	0.17%

Total absorption	0.088	0.0031	70.92%	70.92%	0.17%

*In vitro* data obtained from GSH dosage in the experimental model are reported. The applied and measured GSH amounts are expressed as mg (mean ± standard deviation SD) in the collected medium at different times and homogenized tissue at the end of experimental period. The (%) absorption values, calculated on GSH applied amount, both partial absorption related to each single monitored experimental time and total absorption (the sum of the % in the consecutive times), are calculated and reported in the table too.

**Table 3:** MTT toxicity test.

	OD 540	Tissue viability	Classification
Untreated tissue	1.486	100%	
Treated tissue	1.476	99.33%	NI

*In vitro* data obtained from the MTT irritation test performed on the HOE tissue for the evaluation of tissue viability after tested product treatment.

**Table 4:** GSH level.

	Absorption times		
	Basal	T30	T60
	(nmol/g Hb)	(nmol/g Hb)	(nmol/g Hb)
Mean	7358	8502	8913
SD	1590	1303	1309

*p* value	0.014 (*∗*)		

*In vivo* data obtained from the GSH dosage in total blood are reported. Results are expressed as mean ± SD and expressed in nmol GSH per gram of Hb at different experimental times T30 and T60 versus basal (before starting orobuccal fast-slow release tablet absorption). Statistical analysis was carried out using repeated measures analysis of variance in order to compare the mean values at subsequent times. The GSH level increased significantly (^*∗*^ *p* = 0.014) with absorption time.

## Abbreviations

AD: Alzheimer's diseases
CI: Confidence interval
GR: Glutathione reductase
GSH: Glutathione reduced form
GSSG: Glutathione disulfide oxidized form
GSTs: Glutathione transferases
Hb: Hemoglobin
HOE: Human oral epithelium
HPLC: High-performance liquid chromatography
I: Irritant classification
JNK: Jun kinase
MTT: Methylthiazolyldiphenyl-tetrazolium bromide
NADPH: *β*-Nicotinamide adenine dinucleotide phosphate
NEM: N-ethylmaleimide
NI: Nonirritant classification
OS: Oxidative stress
PD: Parkinson's disease
RDI: Recommended dietary intake
RHE: Reconstructed Human Epithelium
ROS: Reactive oxygen species
SD: Standard deviation of the mean
WMA: Assembly of the World Medical Association.

### In Vitro Times

T10: 10 minutes after product application
T20: 20 minutes after product application
T30: 30 minutes after product application.

### In Vivo Times

T30: 30 minutes starting from tablet absorption
T60: 60 minutes starting from tablet absorption.
